# Hyperacute prediction of functional outcome in spontaneous intracerebral haemorrhage: systematic review and meta-analysis

**DOI:** 10.1177/23969873211067663

**Published:** 2022-02-17

**Authors:** Ulrike Hammerbeck, Aziza Abdulle, Calvin Heal, Adrian R Parry-Jones

**Affiliations:** 1 Geoffrey Jefferson Brain Research Centre, Manchester Academic Health Science Centre, 5292 Northern Care Alliance & University of Manchester, Manchester, UK; 2 Division of Cardiovascular Sciences, Faculty of Biology, Medicine and Health, 12203 University of Manchester, Manchester, UK; 3 School of Physiotherapy, Faculty of Health and Education, 5289 Manchester Metropolitan University, Manchester, UK; 4 Division of Population Health, Faculty of Biology, Medicine and Health, 5292 University of Manchester, Manchester, UK; 5 Manchester Centre for Clinical Neurosciences, Salford Royal NHS Foundation Trust, Salford, UK

**Keywords:** Intracerebral Haemorrhage, functional outcome, predictors

## Abstract

**Purpose:**

To describe the association between factors routinely available in hyperacute care of spontaneous intracerebral haemorrhage (ICH) patients and functional outcome.

**Methods:**

We searched Medline, Embase and CINAHL in February 2020 for original studies reporting associations between markers available within six hours of arrival in hospital and modified Rankin Scale (mRS) at least 6 weeks post-ICH. A random-effects meta-analysis was performed where three or more studies were included.

**Findings:**

Thirty studies were included describing 40 markers. Ten markers underwent meta-analysis and age (OR = 1.06; 95%CI = 1.05 to 1.06; *p* < 0.001), pre-morbid dependence (mRS, OR = 1.73; 95%CI = 1.52 to 1.96; *p* < 0.001), level of consciousness (Glasgow Coma Scale, OR = 0.82; 95%CI = 0.76 to 0.88; *p* < 0.001), stroke severity (National Institutes of Health Stroke Scale, OR=1.19; 95%CI = 1.13 to 1.25; *p* < 0.001), haematoma volume (OR = 1.12; 95%CI=1.07 to 1.16; *p* < 0.001), intraventricular haemorrhage (OR = 2.05; 95%CI = 1.68 to 2.51; *p* < 0.001) and deep (vs. lobar) location (OR = 2.64; 95%CI = 1.65 to 4.24; *p* < 0.001) were predictive of outcome but systolic blood pressure, CT hypodensities and infratentorial location were not. Of the remaining markers, sex, medical history (diabetes, hypertension, prior stroke), prior statin, prior antiplatelet, admission blood results (glucose, cholesterol, estimated glomerular filtration rate) and other imaging features (midline shift, spot sign, sedimentation level, irregular haematoma shape, ultraearly haematoma growth, Graeb score and onset to CT time) were associated with outcome.

**Conclusion:**

Multiple demographic, pre-morbid, clinical, imaging and laboratory factors should all be considered when prognosticating in hyperacute ICH. Incorporating these in to accurate and precise models will help to ensure appropriate levels of care for individual patients.

## Introduction

Spontaneous intracerebral haemorrhage (ICH) accounts for around 10% of incident strokes in Western Europe with an annual worldwide incidence of over three million.^1^ Around a third of patients die within 30 days, often in the first few days.^2^ Many of those that survive remain dependent, with only 20% achieving functional independence at six months.^2^ Globally, it is estimated that 62.8 million disability-adjusted life years are lost annually as a result of ICH.^1^ Despite this major global burden, ICH has few effective treatments.^3^ This, combined with experience of poor outcomes, may lead clinicians to a pessimistic view of prognosis in ICH.^4^

Current treatment for ICH is focussed on rapid reversal of anticoagulation, intensive lowering of blood pressure and surgery for carefully selected cases.^5^ Clinicians may also need to decide, within hours of presentation to hospital, the appropriate level of supportive care, ranging from admission to a critical care unit with full organ support, to care on a stroke or neurosurgical unit, to withdrawal of active care and palliation.^5,6^ This decision is critically dependent on clinicians’ ability to reliably predict the likelihood of a good functional outcome and an overly pessimistic view may lead to inappropriate limitations of care – a self-fulfilling prophecy. Prediction models have been available for the last 20 years and focussed largely on mortality whereas prediction of functional outcome has received less attention.^7–10^ A meta-analysis of Gregorio et al, 2018 found that prediction models perform well for mortality and functional outcome but that the quality of studies was low and the length of outcome too short. With recent evidence that ICH outcomes can be significantly improved with excellent standard care such as anticoagulant reversal, blood pressure lowering and supportive care^11^ and routine performance of new investigations (e.g. Computed Tomography Angiography), it is important to establish whether the current prediction tools are missing important predictors and are well calibrated to outcomes with contemporary stroke care. With the advent of new technology available to clinicians at the bedside (e.g. smartphones and computers), smarter tools with more sophisticated regression models or machine learning algorithms could be developed for improved prognostication to assist optimal decision-making and patient stratification in research studies.

To understand which routinely available factors predict functional outcome during the hyperacute phase of ICH care, we performed a systematic review and meta-analysis describing associations between functional outcome and demographic, pre-morbid, clinical, imaging and laboratory markers available in the first 6 hours after presentation. The strength of this analysis is that it only incorporates studies that have been prospectively collected and adjusted for key confounders of recovery in ICH as proposed in guidelines of performing systematic reviews (Riley et al, 2019).

## Methods

Published guidance for systematic review and meta-analysis of prognostic studies were followed during the design, conduct, analysis and reporting of this work.^12,13^ A PRISMA checklist was completed (Supplementary material) and the protocol prospectively registered [PROSPERO CRD42020159110].

### Eligibility criteria

We included original, observational, randomised control or non-randomised control trials, performed in human adults aged 18 and over with primary ICH. Studies were eligible if participants received standard medical treatment, or no treatment and routine markers were collected within six hours of hospital arrival. Functional outcome had to be measured by the modified Rankin Scale (mRS) at least 6 weeks after admission. As an additional quality measure, the mRS had to be collected prospectively. Studies were excluded if the ICH was caused by tumour, vascular malformation or aneurysm or if participants underwent surgical interventions. In accordance with published guidance,^13^ we excluded studies which did not adjust for key prognostic factors in ICH, (namely age, Glasgow Coma Scale (GCS) or other severity score, ICH volume, ICH location, the presence of secondary intraventricular haemorrhage (IVH) and the prior use of anticoagulants).^8^

### Search strategy and study selection

An electronic database search was performed on 10^th^ February 2020 in; MEDLINE®, Embase and CINAHL plus (see Supplementary material) with search strings based on concepts of: ‘intracerebral h$emorrhage’, ‘predict*’ and ‘function* outcome*’. Reference lists of included studies were scanned for additional studies. Searches were restricted to English publications. The titles, abstracts and full texts were screened consecutively for inclusion by two independent assessors (AA and UH). Agreement was resolved by discussion, or a third assessor (AP-J). If studies contained the same patient cohort and marker, the study with the larger patient cohort was included.

### Data extraction and quality assessment

We adapted the CHARMS-PF checklist^13^ as data extraction tool. Data were independently extracted by two authors (AA and UH), compared and inconsistencies resolved by consensus.

### Risk of bias assessment

Study quality was assessed by two authors using the Quality in Prognosis Studies tool (QUIPS).^13^ A third author, a statistician (CH), performed the risk of bias assessment for statistical analysis and reporting.

### Data synthesis and analysis

We categorised markers into demographic, pre-morbid health, clinical presentation and laboratory findings or findings/features on computer tomography (CT). Meta-analyses were performed for markers with data from three or more studies. For each marker the effect size, in the form of the adjusted odds ratio (OR) with corresponding 95% confidence interval (CI 95%) was extracted. We established the association of these markers with poor outcome and included studies that reported poor outcome as mRS ≥3 and mRS≥4. Studies reporting the ORs for ‘good’ outcome were converted. If mRS could not be converted to this binary cut-off, (e.g., ordinal logistic regression), the findings were only included in the narrative review. We analysed the effect of Hounsfield unit heterogeneity in the haematoma by grouping studies that described areas of low CT attenuation values,^14–16^ or the combined effect of low attenuation, blend sign and black hole sign.^17^ We refer to these combined factors as ‘CT hypodensities’. The statistics for the combined effect of these features were provided by the authors upon request. The effect of ICH location was analysed in categories of deep (supratentorial) vs lobar and infratentorial vs lobar location.

Heterogeneity was assessed using the I^2 statistic^18^ with values <25% considered low and >80% high with *p* < 0.1 deemed significant^18^. Due to high heterogeneity of included studies, we used a random-effects approach for the primary analysis.^18^ A fixed-effect meta-analysis was used for sensitivity analysis (Supplementary material). All analysis was performed using Stata version 14.

### Findings

**Figure 1.**
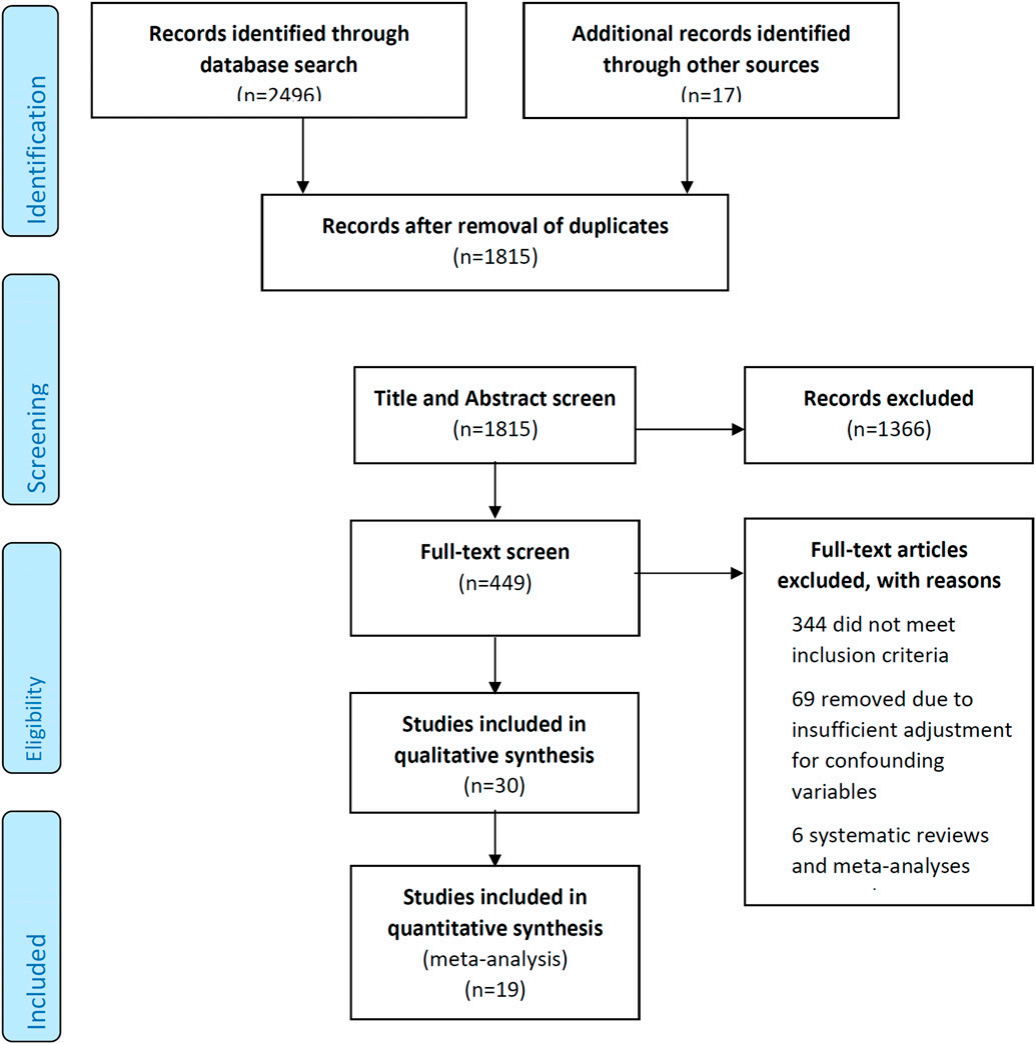
PRISMA flow chart of study selection.

The electronic database search generated 2496 results (Figure 1). After elimination of duplicates and a separate title and abstract screen, 449 studies remained for full text screen. 344 did not meet inclusion criteria, 69 did not adjust for key prognostic factors and 6 were systematic reviews. Eliminating these 419 studies, 30 studies remained^14–17,19–44^, describing 40 markers.

A summary of the included studies is presented in Supplementary material. The 30 included studies consisted of eight prospective cohort studies,^19,20,22,24,29,34,37,44^ 22 retrospective analyses of cohorts with prospective outcome measurement collection^14–17,21,23,25–28, 30–33,35,36,38–43^, and eight studies of cohorts from randomised controlled trials.^15,17,32,33,38,39,42,43^

## Risk of bias

Table 1: Information of included studies (cont). ACS= acute coronary syndrome, BP = blood pressure, CAD= Coronary artery disease, CSS= Canadian stroke scale, , DNR= Do not resuscitate, DWI= diffusion weighted imaging, eGFR= estimated glomerular filtration rate, GCS= Glasgow Coma Score, HR= Heart rate, HTN= Hypertension, ICH-V= ICH volume, ICH-L= ICH Location, MAP= mean arterial blood pressure, NIHSS= National institute of health stroke severity scale, NLR= Neutrophil: Lymphocyte, SBP= Systolic blood pressure, SAH= Subarachnoid haemorrhage, uHG= ultraearly haematoma growth, WMH= white matter hyperintensities

Table 1: Information of included studies. ACS= acute coronary syndrome, BP= blood pressure, CAD= Coronary artery disease, CSS= Canadian stroke scale, , DNR= Do not resuscitate, DWI= diffusion weighted imaging, eGFR= estimated glomerular filtration rate, GCS= Glasgow Coma Score, HR= Heart rate, HTN= Hypertension, ICH-V= ICH volume, ICH-L= ICH Location, MAP= mean arterial blood pressure, NIHSS= National institute of health stroke severity scale, NLR= Neutrophil: Lymphocyte, SBP= Systolic blood pressure, SAH= Subarachnoid haemorrhage, uHG= ultraearly haematoma growth, WMH= white matter hyperintensities

**Figure 2.**
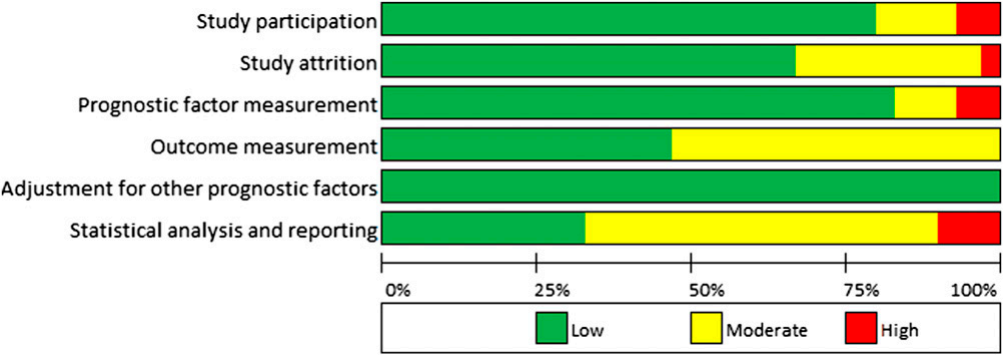
QUIPS (20) risk of bias summary

In general, the risk of reporting bias was low to moderate in the included studies with the highest risk observed in the reporting of outcome measurement and statistical analysis. In both categories over 50% of studies had moderate to severe risk of reporting bias (Figure 2 and Supplementary material). As adjustment for key prognostic factors was an inclusion criterion, this bias was negated.

## Meta-analysis

### Patient demographics and clinical factors

**Figure 3.**
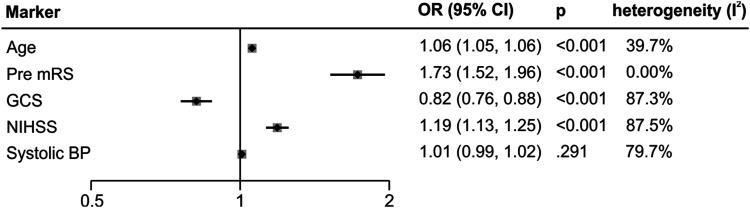
Meta-analysis of association between poor outcome and patient characteristics and clinical presentation.

Meta-analysis of patient characteristics (Figure 3& Supplementary material for raw data) confirmed a strong association between poor outcome and increasing age (OR: 1.06 95% confidence interval (CI): 1.05 to 1.06 *p* < 0.001)^14,16,17,19,23,25,26,28,29^, pre-morbid dependence (mRS, OR = 1.73 CI = 1.52 to 1.96 *p* < 0.001)^17,30,31^ and greater stroke severity in respect to consciousness (GCS, OR= 0.82 CI = 0.76 to 0.88 *p* < 0.001)^14,16,17,22,25,26,28,29,31^ and impairment (National Institute of Health Stroke Scale (NIHSS) score, OR= 1.19 CI =1.13 to 1.25 *p* < 0.001)^20,21,23,24, 26, 31^. In our dataset, we did not find conclusive evidence that systolic blood pressure (SBP, OR= 1.01 CI = 0.99 to 1.02 *p* = 0.29)^17, 29, 30^ was associated with outcome. Study heterogeneity was high for GCS, NIHSS and SBP analysis.

### CT scan features

**Figure 4.**
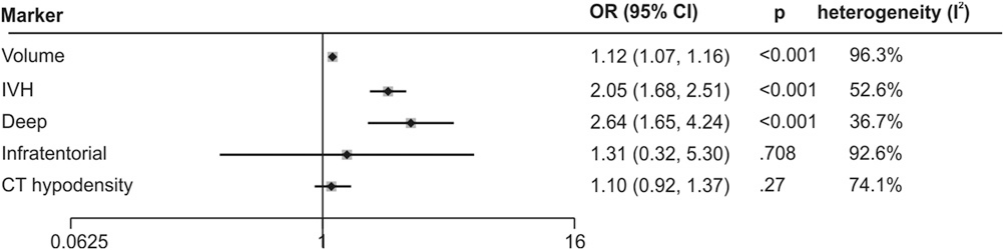
Meta-analysis of association between poor outcome and features on CT.

Meta-analysis confirmed that greater ICH volume (OR= 1.12 CI= 1.07 to 1.16 *p* < 0.001)^14,16,17,22–26,28,29,31^, the presence of IVH (OR= 2.05 CI = 1.68 to 2.51 *p* < 0.001)^14,16,17,19,21,24–26,28,29,31,34^ and deep (vs. lobar) location of the bleed (OR= 2.64 CI=1.65 to 4.24 *p* < 0.001)^16,22,25,27,44^ are predictors of poor functional outcome (Figure 4 Supplementary material for raw data). Our meta-analysis did not find an association of either CT hypodensity (OR = 1.1 CI = 0.92 to 1.37 *p* = 0.27)^14–17^ or infratentorial haemorrhage location (OR = 1.31 CI = 0.32 to 5.30 *p* = 0.71)^14,16,25,27^ with outcome. When sub-dividing infratentorial location, neither cerebellar ICH (vs. lobar, OR = 0.58 CI = 0.07 to 4.49 *p* = 0.6),^19,25,27^ nor brainstem ICH (vs. lobar, OR = 6.05 CI = 0.87 to 41.94 *p* = 0.07)^25,27^ were predictive of poor functional outcome. Study heterogeneity was high for ICH volume and CT hypodensities and medium for the other markers. Although midline shift was reported in three studies,^19,22,31^ data from one study could not be included due to a clear data reporting error^22^ precluding the marker from meta-analysis.

## Narrative review

### Patient demographics, clinical and laboratory factors

Other demographic and pre-morbid factors associated with poor outcome were female sex,^17,28^ previous stroke,^21^ diabetes^39^ and the use of pre-morbid antiplatelet therapy.^17,19^ Results for anticoagulants showed an association with poor outcome in one study^21^ and another showing no association.^16^ Laboratory findings associated with a poor outcome were decreased fibrinogen concentration,^20^ moderately reduced estimated glomerular filtration rates (eGFR)^43^ and high admission glucose.^21,26,39,41^ Good outcome was associated with hypocholesterolaemia^31^, lower heart rate^32^ and continued or new statin use.^40^

Other demographic and pre-morbid features including ethnicity (black/Hispanic),^27^ hypertension,^19,21^ coronary artery disease^19^ and smoking history^19^ were not associated with outcome. Presenting factors with no association with outcome included mean arterial BP,^22^ conjugate eye movements,^37^ dyslipidaemia,^19^ increased neutrophil to lymphocyte ratio (NLR)^23^ or increased white blood cell count.^31,42^

### CT scan features

Other factors predictive of poor outcome were midline shift,^19,31^ irregular haemorrhage shape,^15^ ultraearly haematoma growth,^35^ the Graeb score,^36^ presence of the spot sign^24,35^ and increased onset to CT time.^17^ A predictors of good outcome was the absence of a sedimentation level.^38^ The hemisphere of the ICH^22^ and associated subarachnoid haemorrhage (SAH)^29^ were not predictive of outcome.

## Discussion

We systematically reviewed the literature for the association between demographic, clinical, imaging and laboratory factors routinely available within six hours of onset and functional outcome in ICH. Our inclusion criteria sought to identify only high-quality studies where adequate adjustment for cofactors was made and outcomes were prospectively collected. For ten markers, three or more studies were included which allowed meta-analysis. We found that increasing age, pre-morbid dependence (mRS), depressed consciousness (GCS), greater impairment (NIHSS score) as well as imaging markers of greater ICH volume, IVH and deep (vs. lobar) location were clearly associated with worse functional outcome. All of these are established prognostic factors which are included in previously described predictive models, except for pre-morbid mRS^7–10^.

### Demographic, pre-morbid and baseline clinical factors

Although our meta-analysis confirms that pre-morbid mRS is strongly associated with functional outcome, it is not included in any previously described prognostic model.^8^ Inclusion of pre-morbid mRS in future predictive models may improve their performance and should be further evaluated. In two studies, female sex was independently associated with poor outcome. This could be because advancing age is a stronger predictor of poor outcome in females^45^ and recent evidence in ischaemic stroke demonstrates that women have worse functional outcomes and quality of life, partly explained by variations in management.^46^ Other factors relating to risk of ICH, baseline features, pathophysiology, response to treatment and management decisions may all play a role and warrant further investigation.^47^

We found no clear association between functional outcome and acute blood pressure parameters or hypertension^19,21^. Blood pressure is included in many prognostic models for mortality but not morbidity.^8^ Any association between acute BP parameters and outcome may be driven by co-linearity with ICH severity, thus only including studies with adequate baseline adjustment may explain the lack of association. Furthermore, a recent meta-analysis has suggested that intensive blood pressure lowering may reduce haematoma growth but does not seem to improve functional outcome^48^, lending further support to our findings. However, with our search criteria, insufficient studies^17,22,29,30^ were included in the SBP and MAP analysis to make firm conclusions and differences in defining hypertension made pooling of datasets difficult.^19,21^

### Medication use

We identified an association between prior antiplatelet use and poor outcome in two studies. A recent meta-analysis found a non-statistically significant (*p* = 0.06) trend towards worse outcome^49^ but included studies without requirements for minimal adjustment for cofactors. In contrast, only one of the two studies describing prior anticoagulant use showed an association with poor functional outcome, with no association seen in the other^16,21^ Whilst anticoagulants are associated with larger baseline haematomas,^50^ this has been adjusted for in the included studies. Changing patterns of anticoagulant use and subsequent reversal treatment may further serve to modify relationships with outcome and will require further investigation. Continuing or commencing a statin^40^ during admission was associated with good functional outcome compared to no statin use, but the association may be driven by physicians’ termination of use, if a poor outcome is anticipated, rather than a causal effect.^40^ It is of note, however, that we also found hypercholesterolaemia to be associated with good outcome,^31^ but interpretation is complicated, as patients taking cholesterol lowering medication were classed as having hypercholesterolaemia regardless of laboratory results. Randomised controlled trials of statins in ICH will be required to determine the benefits of statins on outcome, such as the ongoing SATURN trial^51^.

### Laboratory results

Along with hypercholesterolaemia, an independent association between poor outcome and lower eGFR and fibrinogen and higher glucose was found. Low fibrinogen may be associated with ongoing bleeding and haematoma expansion and treatment with fibrinogen concentrate has been proposed as a possible therapy.^52^ Elevated glucose is a well-established marker of poor prognosis in stroke, with guidelines recommending treatment^53^ to maintain tight control.

### Imaging features

The limited data included in the meta-analysis did not demonstrate an association of infratentorial haemorrhage location and functional outcome. This could be explained by grouping brainstem and cerebellum ICH with very different clinical characteristics and prognoses.^54^ Insufficient studies were included for definitive answers but in post hoc analyses, we found no association between poor outcome and cerebellar ICH^19,25,27^ and only a trend towards poor outcome in brainstem ICH.^25,27^ The lack of an accepted consensus approach to defining location hampered our analysis, but the recently described CHARTS instrument seeks to address this.^55^

Haematoma hypodensities on CT scans are thought to identify ongoing bleeding and thereby can identify individuals at risk of haematoma expansion.^16^ To facilitate meta-analysis, we combined studies reporting hypodensities,^14-17^ black hole and blend signs^17^ as indicators of unclotted blood in the haematoma. Whilst individual studies found specific signs to be associated, hypodensities as a group were not associated with outcome. Furthermore, the methods to define hypodensity may be important, for example using a specific Hounsfield unit threshold of 32,^14^ rather than subjective reporting of the presence or absence of hypodensities. Additional factors that were predictive of poor outcome on imaging but with insufficient studies for inclusion in the meta-analysis were midline shift, irregular haematoma shape, ultraearly haematoma growth, Graeb score and onset to CT time. In contrast, the side of the ICH or additional SAH did not predict a poor outcome.

### Predicting modified mRS at 90-days

We included studies using the mRS^56^ at or after 6 weeks post-stroke to enable meta-analysis. The mRS is a robust, commonly used measure of functional outcome, including death, in clinical practice and in clinical trials.^57^ It can be assessed over the phone and thereby minimises loss to follow-up.^58^ However, it only crudely measures functional status by the amount of assistance required for daily activity. In the included studies, the mRS was predominantly reported as a binary measure. The definition of ‘poor outcome’ was however not consistent and we included and combined studies that classified poor outcome either as an mRS of 4–6 or 3–6, as has been done in previous meta-analyses.^8^ Most included studies reported functional outcome at 90 days but improvements in outcome have been observed up to a year after ICH.^7,59^ Further work should establish whether predictors differ for outcomes beyond 90 days.

### Strength and limitations

Specific strengths of this review are a broad inclusive search strategy and a narrow focus on routinely available factors during acute hospital admission. We additionally only included studies reporting an odds ratio adjusted for at least age, GCS, ICH lesion volume, ICH location, IVH and prior anticoagulation and where outcome was prospectively collected.

There are a few limitations to this review. It is interesting that none of the included papers investigated the effect of the presence or severity of small vessel disease, on outcome. We excluded studies where surgical interventions were performed but acknowledge that there is a possibility that after study inclusion, surgical interventions could be performed in some subjects without being reported. As frequently observed in systematic review of prognostic studies,^13^ study heterogeneity was high, and the risk of bias observed in our study is increased due to reduced detail regarding loss to follow-up. The heterogeneity could partly stem from the wide variety of confounders that different studies have adjusted for (Supplementary material) with a resultant risk of a reduction of power.

## Conclusion

Demographic, pre-morbid, clinical, imaging and laboratory factors should all be considered when estimating prognosis in hyperacute ICH. Accurate and precise models incorporating such factors will help to ensure appropriate care for individual patients, reducing the self-fulfilling prophecy of an overly pessimistic prognosis and conversely, ensuring that appropriate palliative care is provided to those with little or no chance of survival with a reasonable quality of life. Given ubiquitous access to electronic devices at the point of care along with electronic patient records, more complex scores incorporating all predictive factors should be developed and validated and could be easily incorporated into clinical care pathways. Fifteen previously reported prognostic models for functional outcome have a median of 5 factors included with a range of 2 to 8, yielding a C-statistic ranging from 0.81 to 0.93 ^8^. Whether the addition of all factors identified in our review would improve the prognostication and thereby patient outcome would need to be established in large prospectively collected cohorts.

## Supplemental Material

sj-pdf-1-eso-10.1177_23969873211067663 – Supplemental Material for Hyperacute prediction of functional outcome in spontaneous intracerebral haemorrhage: systematic review and meta-analysis Click here for additional data file. Supplemental Material, sj-pdf-1-eso-10.1177_23969873211067663 for Hyperacute prediction of functional outcome in spontaneous intracerebral haemorrhage: systematic review and meta-analysis by Ulrike Hammerbeck, Aziza Abdulle, Calvin Heal and Adrian R Parry-JonesPaul in European Stroke Journal
