# Supplementary material for: Hyperacute prediction of functional outcome in spontaneous intracerebral haemorrhage: systematic review and meta-analysis
Source: Eur Stroke J. 2022 Feb 17;7(1):6–14. doi: 10.1177/23969873211067663 (PMC8921779; doi:10.1177/23969873211067663)
Supplement: sj-pdf-1-eso-10.1177_23969873211067663 – Supplemental Material for Hyperacute prediction of functional outcome in spontaneous intracerebral haemorrhage: systematic review and meta-analysis [file sj-pdf-1-eso-10.1177_23969873211067663.pdf]

Title: Hyperacute prediction of long-term functional outcome in spontaneous intracerebral haemorrhage: systematic review and meta-analysis

Supplementary figures and tables

Supplementary Figure1: Sensitivity analysis: Fixed effect meta-analysis of association between poor outcome and patient characteristics and presenting symptoms.

Supplementary Figure1: Sensitivity analysis: Fixed effect meta-analysis of association between poor outcome and features on CT.

Supplementary Table 1: PRISMA Checklist
Supplementary Table 2: Search strategy
Supplementary Table 3: Study details
Supplementary Table 4: Risk of Bias: Individual study analysis
Supplementary Table 5: Data included for each factor: Clinical
Supplementary Table 6: Data included for each Factor: CT

Supplementary files

Supplementary Figure 1: Fixed effect meta-analysis of association between poor outcome and patient characteristics and presenting symptoms.

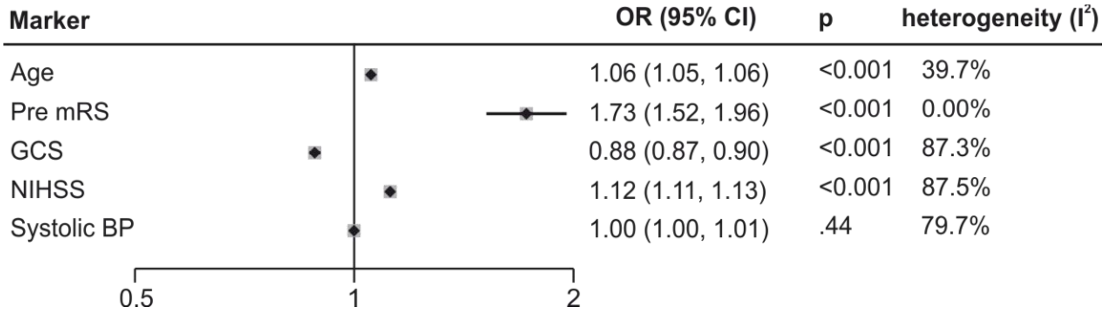

Supplementary Figure 2: Sensitivity analysis: Fixed effect meta-analysis of association between poor outcome and features on CT.

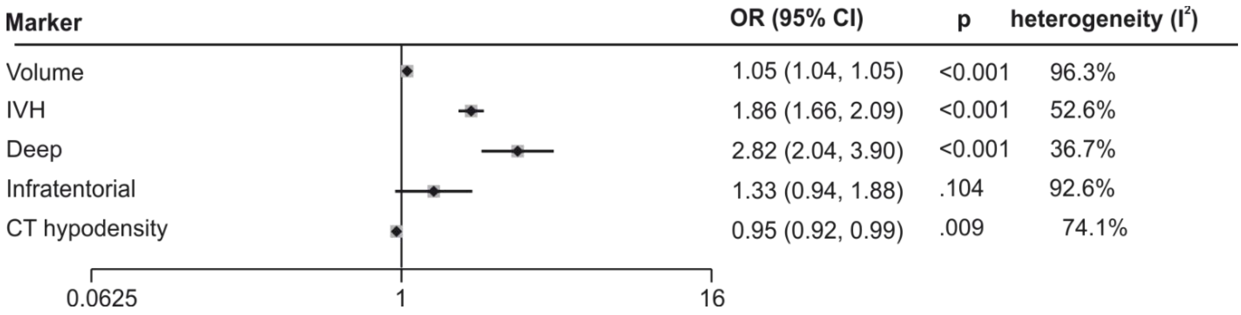

Supplementary table 1: PRISMA checklist

Section/topic	#	Checklist item	Reported on page #
**TITLE**			
Title	1	Identify the report as a systematic review, meta-analysis, or both.	1
**ABSTRACT**			
Structured summary	2	Provide a structured summary including, as applicable: background; objectives; data sources; study eligibility criteria, participants, and interventions; study appraisal and synthesis methods; results; limitations; conclusions and implications of key findings; systematic review registration number.	2
**INTRODUCTION**			
Rationale	3	Describe the rationale for the review in the context of what is already known.	3
Objectives	4	Provide an explicit statement of questions being addressed with reference to participants, interventions, comparisons, outcomes, and study design (PICOS).	3?
**METHODS**			
Protocol and registration	5	Indicate if a review protocol exists, if and where it can be accessed (e.g., Web address), and, if available, provide registration information including registration number.	4
Eligibility criteria	6	Specify study characteristics (e.g., PICOS, length of follow-up) and report characteristics (e.g., years considered, language, publication status) used as criteria for eligibility, giving rationale.	4
Information sources	7	Describe all information sources (e.g., databases with dates of coverage, contact with study authors to identify additional studies) in the search and date last searched.	4
Search	8	Present full electronic search strategy for at least one database, including any limits used, such that it could be repeated.	4
Study selection	9	State the process for selecting studies (i.e., screening, eligibility, included in systematic review, and, if applicable, included in the meta-analysis).	4

Data collection process	10	Describe method of data extraction from reports (e.g., piloted forms, independently, in duplicate) and any processes for obtaining and confirming data from investigators.	4
Data items	11	List and define all variables for which data were sought (e.g., PICOS, funding sources) and any assumptions and simplifications made.	4
Risk of bias in individual studies	12	Describe methods used for assessing risk of bias of individual studies (including specification of whether this was done at the study or outcome level), and how this information is to be used in any data synthesis.	5
Summary measures	13	State the principal summary measures (e.g., risk ratio, difference in means).	5
Synthesis of results	14	Describe the methods of handling data and combining results of studies, if done, including measures of consistency (e.g., I^2) for each meta-analysis.	5

Risk of bias across studies	15	Specify any assessment of risk of bias that may affect the cumulative evidence (e.g., publication bias, selective reporting within studies).	?
Additional analyses	16	Describe methods of additional analyses (e.g., sensitivity or subgroup analyses, meta-regression), if done, indicating which were pre-specified.	5
**RESULTS**			
Study selection	17	Give numbers of studies screened, assessed for eligibility, and included in the review, with reasons for exclusions at each stage, ideally with a flow diagram.	6
Study characteristics	18	For each study, present characteristics for which data were extracted (e.g., study size, PICOS, follow-up period) and provide the citations.	?
Risk of bias within studies	19	Present data on risk of bias of each study and, if available, any outcome level assessment (see item 12).	6
Results of individual studies	20	For all outcomes considered (benefits or harms), present, for each study: (a) simple summary data for each intervention group (b) effect estimates and confidence intervals, ideally with a forest plot.	
Synthesis of results	21	Present results of each meta-analysis done, including confidence intervals and measures of consistency.	
Risk of bias across studies	22	Present results of any assessment of risk of bias across studies (see Item 15).	?
Additional analysis	23	Give results of additional analyses, if done (e.g., sensitivity or subgroup analyses, meta-regression [see	

		Item 16]).	
**DISCUSSION**			
Summary of evidence	24	Summarize the main findings including the strength of evidence for each main outcome; consider their relevance to key groups (e.g., healthcare providers, users, and policy makers).	
Limitations	25	Discuss limitations at study and outcome level (e.g., risk of bias), and at review-level (e.g., incomplete retrieval of identified research, reporting bias).	
Conclusions	26	Provide a general interpretation of the results in the context of other evidence, and implications for future research.	
**FUNDING**			
Funding	27	Describe sources of funding for the systematic review and other support (e.g., supply of data); role of funders for the systematic review.	

Supplementary Table 2: Search Strategy

**Medline (Ahead of Print, In-Process & Other Non-Indexed Citations, Daily and Versions(R) 1946 to February 07, 2020)**	(‘Cerebral hemorrhage’) AND (‘Predict*’) AND (‘Recovery of Function’ OR ‘function* outcome*’ OR ‘recover* function*’ OR ‘outcome’)
**Embase (1974 to 2020 February 07)**	(‘Intracerebral H$emorrhage*’ OR ‘Cerebral h$emorrhage*’) AND (‘recover* of function*’ OR ‘function* outcome*’) AND (‘predict*’)
**CINAHL plus**	(‘Cererbral Hemorrhage’ OR ‘intracerebral haemorrhage’ OR ‘intracerebral hemorrhage’). AND<br>(‘Functional Status’ OR ‘recover* of function*’ OR ‘function* outcome*’ OR ‘outcome*’) AND<br>(‘Predictive Research’ OR ‘predict*’)

Supplementary Table 3: Study details

	Study design	Study population	Study Duration	Sample	Predictive values	Follow-up	mRS cut-off		Additional adjusting confounding variable (in addition to age, GCS, ICH location, volume, IVH, anticoagulant use)
**Asadollahi et al, 2016(1)**	Prospective	Iran	2011-2012	228	Age, Antiplatelets, CAD, Dyslipidaemia, HTN, ICH-L, ICH-V, IVH, Midline shift, Smoking	3 years	4	M/N	Antiplatelets, Dyslipidaemia, CAD, HTN, ICH score, Midline shift, Smoking
**Boulouis et al, 2016(2)**	Retrospective	USA	1994-2016	800	Age, CT hypodensities, GCS, ICH-L, ICH-V, IVH, Warfarin	3 months	4	M/N	
**Castellanos et al, 2005(3)**	Retrospective	Spain	1999-2001	138	CSS, Fibrinogen levels, ICH Location	3 months	3	M/N	Fibrinogen levels
**Chu et al, 2019(4)**	Retrospective	China	2011-2014	311	Age, GCS, ICH-L, ICH-V, IVH, Minimal CT attenuation value	3 months	4	M	Minimal CT attenuation value (+/ </31 HU)
**Delcourt et al, 2016(5)**	RCT	Multiple	2008-2012	2066	Haematoma density, Haematoma Shape	3 months	3	M/N	Antiplatelets, China, Decision to withdraw treatment , Onset to CT scan time, Randomized treatment, SBP
**Dowlatshahi et al, 2011(6)**	Retrospective	Multiple	unknown	496	Age, Anticoagulant use, Blood glucose, ICH-V, IVH, NIHSS, Prior HTN, Prior stroke	3 months	4	M/N	Antiplatelets, Blood glucose, BP, Sex, Onset-to-CT time, Smoking
**El-Senousey et al, 2010(7)**	Prospective	Egypt	Unknown	67	Age, GCS, ICH-L, ICH side, ICH-V, IVE, MAP, Midline shift	2 months	4	M/N	ICH side, MAP, Midline shift

	Study design	Study population	Study Duration	Sample	Predictive values	Follow-up	mRS cut-off		Additional adjusting confounding variable (in addition to age, GCS, ICH location, volume, IVH, anticoagulant use)
**Giede-Jeppe et al, 2017(8)**	Retrospective	Germany	2006-2014	855	Age, ICH-V, NIHSS, NLR	3 months	4	M/N	Dyslipidaemia, Graeb score, Haemoglobin, Haematocrit, ICH score, Leucocytes, MAP, Midline shift, Pre-morbid mRS
**Havesteen et al, 2014(9)**	Prospective	Denmark	2009-2013	128	Age, ICH-V, NIHSS, Spot sign	3 months	5	M/N	Blood glucose, Sex, Pre-morbid mRS, SAH, Spot sign
**Ironside et al, 2019(10)**	Retrospective	USA	2009-2017	311	Age, GCS, ICH-L, ICH-V, IVH	3 months	2	M	
**Ji et al, 2013(11)**	Retrospective	China	2007-2008	3255	Age, Blood glucose, GCS, ICH-L, ICH-V, IVH, NIHSS	1 year	3	M/N	Antiplatelets, Sex, Hospital academic status, Laboratory tests on admission, Statins, Stroke risk factors, Transportation mode to hospital
**Kidwell et al, 2017(12)**	Prospective	USA	2011-2014	600	ICH-L, Race	3 months	4	M/N	DWI lesion count, In hospital HTN treatment, Race, WMH score
**Law et al, 2020(13)**	Retrospective	Multiple	2013-2017	2307	Age, Antiplatelets, Blend sign, Blackhole sign, GCS, Sex, Hypodensities, ICH-L, ICH-V, Island sign, IVH, Onset-to-CT, Pre-morbid mRS, SBP, Tranexamic acid	3 months	4	M/N	Antiplatelet, Black hole sign, Blend sign, Hypodensities, Island sign, Onset-to-CT, Pre-morbid mRS, SBP, Sex, Tranexamic acid,
**Leasure et al, 2019(14)**	Retrospective	USA	2011-2013	2139	Age, GCS, Sex, ICH-V, IVH	3 months	4	M/N	Sex
**Li et al, 2017(15)**	Prospective	China	2011-2016	252	Age, GCS, ICH-L, ICH-V, Island sign, IVH, SAH, SBP	3 months	3	M/N	Alcohol, Diabetes, HTN, Island sign, SAH, SBP, Smoking

	Study design	Study population	Study Duration	Sample	Predictive values	Follow-up	mRS cut-off		Additional adjusting confounding variable (in addition to age, GCS, ICH location, volume, IVH, anticoagulant use)
**Li et al, 2018(16)**	Retrospective	china	2011-2016	225	ICH-L	3 months	4	M	Black hole sign
**Miyares et al, 2020(17)**	Retrospective	USA	2011-2015	418	Pre-morbid mRS, SBP	3 months	4	M	Male, Pre-morbid mRS, Race, SBP
**Palm et al, 2013(18)**	Retrospective	Germany	2006-2010	152	GCS, Hypercholesterolaemia, ICH-V, IVH, Leukocyte count, Midline shift, NIHSS, Prior mRS,	1 year	4	M/N	GCS, NIHSS, DNR order, Hypercholesterolemia, Pre-morbid mRS, ICH-V, Midline shift, IVH Leukocyte count, Age, Sex
**Qiu et al,, 2016(19)**	Retrospective	Multiple	2005-2012	3185	HR	3 months	Shift of 1	N	Antiplatelets, β-blockers, China, Female, Intensive BP-lowering treatment, SBP, Time from onset to randomization
**Rådholm et al, 2015(20)**	RCT	Multiple	2008-2012	2794	Age	3 months	3	N	Anti-HTN drugs, Blood glucose, Diabetes, Prior stroke, Recruitment from china, Randomised treatment, Sex, SBP, time from ICH onset to baseline CT,
**Rodriguez-Luna et al, 2011(21)**	Prospective	Spain	2009-2010	108	Age, ICH-V, IVH, NIHSS	3 months	3	M/N	Albumin, Blood glucose, Body temperature, Statin
**Rodriguez-Luna et al, 2016(22)**	Retrospective	Multiple	2006-2010	178	ICH-V, Spot sign, uHG,	3 months	3	N	Sex

	Study design	Study population	Study Duration	Sample	Predictive values	Follow-up	mRS cut-off		**Additional adjusting confounding variable** (in addition to age, GCS, ICH location, volume, IVH, anticoagulant use)
**Roeder et al, 2019(23)**	Retrospective	Germany	2006-2015	1112	Graeb score	3 months	4	N	Pre-morbid mRS
**Sato et al, 2012(24)**	Prospective	Japan	2009-2011	211	Conjugate eye deviation	3 months	3	N	Sex
**Sato et al, 2016(25)**	Retrospective	Multiple	2008-2012	2065	Sedimentation level	3 months	3	N	China, Female, onset to CT time, randomized intensive BP lowering,
**Saxena et al, 2016(26)**	RCT	Multiple	2008-2012	2635	Blood glucose, Diabetes	3 months	3	N	Aspirin, Diabetes, Heart disease, HTN, Randomized treatment, Region, SBP, Sex
**Siddiqui et al, 2017(27)**	Retrospective	USA	2011-2013	1093	Statin	3 months	n/a	N	Antiplatelets, pre-morbid mRS, Race, Sex
**Sun et al, 2016(28)**	Retrospective	China	2007-2008	2951	Blood glucose	3 months	3	N	Admitted department, AF, CAD, Craniotomy, Dehydrant agents treatment, Gender, HTN, Pre-morbid mRS, Smoking, Support withdrawal
**Yu et al, 2016(29)**	RCT	Multiple	2008-2012	2630	Leucocyte count	3 months	3	N	Chinese, Blood glucose, Body temperature, HR, Lipid lowering agent, Onset to CT time, Randomized treatment, SBP, Sex
**Zheng et al, 2016(30)**	Retrospective	Multiple	2008-2012	2623	eGFR	3 months	3	N	ACS, Antiplatelets, Chinese, Diabetes, HTN, Ischemic stroke, HTN, Randomly assigned group, Statins, SBP, Time from onset-to-Randomization

Supplementary Table 4: Risk of bias assessment: individual study analysis

	Study participants	Study attrition	Prognostic factor measurements	Outcome measurements	Adjustment for other prognostic factors	Statistical analysis and reporting
**Asadollahi et al, 2016(1)**	Low	Low	Low	Low	Low	High
**Boulouis et al, 2016(2)**	Low	Medium	Low	Low	Low	Medium
**Castellanos et al, 2005(3)**	Low	Low	Low	Medium	Low	Medium
**Chu et al, 2019(4)**	Low	Low	Low	Low	Low	Medium
**Delcourt et al, 2016(5)**	Low	Low	Medium	Medium	Low	Low
**Dowlatshahi et al, 2011(6)**	High	Medium	Medium	Medium	Low	Medium
**El-senousey et al, 2010(7)**	High	Low	High	Medium	Low	Medium
**Giede-Jeppe et al, 2017(8)**	Low	Medium	Low	Low	Low	High
**Havsteen et al, 2014(9)**	Medium	Low	Low	Low	Low	Medium
**Ironside et al, 2019(10)**	Low	Low	Low	Medium	Low	Low
**Ji et al, 2013(11)**	Low	Low	Low	Low	Low	Low
**Kidwell et al, 2017(12)**	Low	High	Low	Low	Low	Low
**Law et al, 2020(13)**	Low	Low	Low	Low	Low	Low
**Leasure et al, 2019(14)**	Low	Medium	Low	Low	Low	Low

**Li et al, 2017(15)**	Low	Mediu m	Low	Medium	Low	Low
**Li et al, 2018(16)**	Medium	Mediu m	Low	Medium	Low	Medium
**Miyares et al, 2020(17)**	Medium	Mediu m	Low	Low	Low	Medium
**Palm et al, 2013(18)**	Low	Mediu m	Low	Medium	Low	Medium
**Qui et al, 2016(19)**	Low	Low	Low	Medium	Low	Medium
**Rådholm et al, 2015(20)**	Low	Low	Low	Medium	Low	Medium
**Rodriguez-Luna et al, 2011(21)**	Medium	Low	Low	Medium	Low	High
**Rodriguez-Luna et al, 2016(22)**	Low	Low	Medium	Medium	Low	Medium
**Roeder et al, 2019(23)**	Low	Low	Low	Low	Low	Low
**Sato et al 2012(24)**	Low	Low	High	Low	Low	Medium
**Sato et al , 2016(25)**	Low	Low	Low	Medium	Low	Medium
**Saxena et al, 2016(26)**	Low	Low	Low	Medium	Low	Medium
**Siddiqui et al, 2017(27)**	Low	Mediu m	Low	Low	Low	Medium
**Sun et al, 2016(28)**	Low	Low	Low	Low	Low	Medium
**Yu et al, 2016(29)**	Low	Low	Low	Medium	Low	Low
**Zheng et al, 2016(30)**	Low	Low	Low	Medium	Low	Low

Supplementary Table 5: Data included for each factor: Clinical factors

Factor	Contributing papers	n	Odds-ratio	CI low	CI high	p	Heterogeneity (I^2)
**Age**		**9624**	**1.06**	**1.05**	**1.06**	**<0.001**	**39.7%**
	Law et al 2020	2307	1.05	1.04	1.06		
	Leasure et al 2019	1305	1.07	1.05	1.08		
	Asadollahi et al 2016	228	1.05	1	1.1		
	Giede-Jeppe et al 2017	855	1.066	1.044	1.088		
	Chu et al 2019	311	1.04	1.001	1.08		
	Ji et al 2013	3255	1.05	1.04	1.06		
	Ironside et al 2019	311	1.074	1.033	1.116		
	Boulouis et al 2016	800	1.07	1.06	1.09		
	Li et al 2017	252	1.04	1.01	1.06		
**Pre-morbid mRS**		**2877**	**1.73**	**1.52**	**1.96**	**<0.001**	**0.00%**
	Law et al 2020	2307	1.71	1.5	1.95		
	Miyares et al 2020	418	16.87	4.86	58.54		
	Palm et al 2013	152	1.98	1.2	3.25		
**GCS**		**8760**	**0.82**	**0.76**	**0.88**	**<0.001**	**87.3%**
	Law et al 2020	2307	0.77	0.72	0.82		
	Chu et al 2019	311	0.598	0.371	0.962		
	Palm et al 2013	152	0.75	0.62	0.92		
	Boulouis et al 2016	800	0.88	0.84	0.92		
	Leasure et al 2019	1305	0.82	0.78	0.86		
	Ji et al 2013	3255	0.92	0.89	0.93		
	Ironside et al 2019	311	0.667	0.548	0.812		
	Li et al 2017	252	0.89	0.8	0.99		
	El-Senousey et al 2010	67	0.19	0.08	0.5		
**NIHSS**		**5024**	**1.19**	**1.13**	**1.25**	**<0.001**	**87.5%**
	Dowlatshahi et al 2011	496	1.2	1.1	1.2		
	Palm et al 2013	152	1.19	1.1	1.3		
	Giede-Jeppe et al 2017	855	1.167	1.13	1.204		
	Ji et al 2013	3255	1.1	1.08	1.11		
	Havesteen et al 2014	128	1.21	1.1	1.32		
	Castellanos et al 2005	138	1.5	1.28	1.77		
**Systolic BP**		**2977**	**1.01**	**0.99**	**1.02**	**0.291**	**79.7%**
	Law et al 2020	2307	1	0.996	1.003		
	Miyares et al 2020	418	1.1	1.02	1.18		
	Li et al 2017	252	1.01	1	1.02		

Supplementary Table 6: Data included for each Factor: CT factors

Factor	Contributing papers	n	Odds-ratio	CI low	CI high	p	Heterogeneity (I^2)
**Volume**		**9743**	**1.12**	**1.07**	**1.16**	**<0.001**	**96.3%**
	Law et al 2020	2307	1.6	1.47	1.74		
	Havesteen et al 2014	128	1.02	1	1.04		
	Palm et al 2013	152	1.03	0.99	1.05		
	Boulouis et al 2016	800	1.56	1.39	1.77		
	Leasure et al 2019	1305	1.09	1.08	1.1		
	Giede-Jeppe et al 2017	855	1.023	1.01	1.037		
	Chu et al 2019	311	1.13	1.027	1.242		
	Ji et al 2013	3255	1.02	1.01	1.03		
	Ironside et al 2019	311	1.375	1.085	1.743		
	Li et al 2017	252	1.04	1.01	1.07		
	El-Senousey et al 2010	67	1.41	1.01	2		
**IVH**		**9778**	**2.05**	**1.68**	**2.51**	**<0.001**	**52.6%**
	Dowlatshahi et al 2011	496	2.3	1.4	3.8		
	Law et al 2020	2307	2.24	1.76	2.87		
	Boulouis et al 2016	800	1.66	1.13	2.47		
	Leasure et al 2019	1305	1.52	1.11	2.07		
	Asadollahi et al 2016	228	3.72	1.16	11.8		
	Chu et al 2019	459	1.424	0.807	1.664		
	Palm et al 2013	129	6.01	2.39	15.12		
	Ji et al 2013	3255	1.62	1.34	1.98		
	Ironside et al 2019	311	2.921	1.076	7.93		
	Li et al 2017	252	3.05	1.61	5.78		
	Rodriguez-Luna et al 2011	108	4.61	1.29	16.49		
	Havesteen et al 2014	128	4.76	1.2	20		
**Deep**		**2003**	**2.64**	**1.65**	**4.24**	**<0.001**	**36.7%**
	Boulouis et al 2016	800	3.1	2	4.9		
	Kidwell et al 2017	600	3.526	1.896	6.558		
	Li et al 2018	225	2.09	0.805	5.45		
	Ironside et al 2019	311	0.934	0.307	2.842		
	El-Senousey et al 2010	67	27.78	0.64	1000		
**Infratentorial**		**2022**	**1.31**	**0.32**	**5.30**	**0.708**	**92.6%**
	Boulouis et al 2016	800	5.17	2.52	8.98		
	Chu et al 2019	311	0.528	0.419	1.122		
	Kidwell et al 2017	600	3.42	1.43	8.16		
	Ironside et al 2019	311	0.24	0.055	1.045		
**Hypodensity**		**4347**	**1.1**	**0.92**	**1.37**	**0.27**	**74.10%**
	Law et al 2020	2307	1.22	0.95	1.56		
	Delcourt et al, 2016	781	1.06	0.85	1.33		
	Boulouis et al 2016	800	1.7	1.1	2.64		
	Chu et al 2019	459	0.945	0.912	0.978		

References

1. Asadollahi S, Vafaei A, Heidari K. CT imaging for long-term functional outcome after spontaneous intracerebral haemorrhage: A 3-year follow-up study. Brain Inj. 2016;30(13-14):1626-34.
2. Boulouis G, Morotti A, Brouwers HB, Charidimou A, Jessel MJ, Auriel E, et al. Noncontrast Computed Tomography Hypodensities Predict Poor Outcome in Intracerebral Hemorrhage Patients. Stroke. 2016;47(10):2511-6.
3. Castellanos M, Leira R, Tejada J, Gil-Peralta A, Dávalos A, Castillo J. Predictors of good outcome in medium to large spontaneous supratentorial intracerebral haemorrhages. J Neurol Neurosurg Psychiatry. 2005;76(5):691-5.
4. Chu H, Huang C, Dong J, Yang X, Xiang J, Mao Y, et al. Minimal Computed Tomography Attenuation Value Within the Hematoma is Associated with Hematoma Expansion and Poor Outcome in Intracerebral Hemorrhage Patients. Neurocrit Care. 2019;31(3):455-65.
5. Delcourt C, Zhang S, Arima H, Sato S, Al-Shahi Salman R, Wang X, et al. Significance of Hematoma Shape and Density in Intracerebral Hemorrhage: The Intensive Blood Pressure Reduction in Acute Intracerebral Hemorrhage Trial Study. Stroke. 2016;47(5):1227-32.
6. Dowlatshahi D, Smith EE, Flaherty ML, Ali M, Lyden P, Demchuk AM. Small Intracerebral Haemorrhages are Associated with Less Haematoma Expansion and Better Outcomes. International Journal of Stroke. 2011;6(3):201-6.
7. El-Senousey MY, Rabie MO, Elbeshlawy WF, Deewan K. Outcome of spontaneous supratentorial intracerebral hematoma. Egyptian Journal of Neurology, Psychiatry and Neurosurgery. 2010;47(2):7.
8. Giede-Jeppe A, Bobinger T, Gerner ST, Sembill JA, Sprügel MI, Beuscher VD, et al. Neutrophil-to-Lymphocyte Ratio Is an Independent Predictor for In-Hospital Mortality in Spontaneous Intracerebral Hemorrhage. Cerebrovascular diseases. 2017;44(1-2):26-34.
9. Havsteen I, Ovesen C, Christensen AF, Hansen CK, Nielsen JK, Christensen H. Showing no spot sign is a strong predictor of independent living after intracerebral haemorrhage. Cerebrovascular diseases. 2014;37(3):164-70.
10. Ironside N, Chen CJ, Dreyer V, Christophe B, Buell TJ, Connolly ES. Location-specific differences in hematoma volume predict outcomes in patients with spontaneous intracerebral hemorrhage. International journal of stroke : official journal of the International Stroke Society. 2020;15(1):90-102.
11. Ji R, Shen H, Pan Y, Wang P, Liu G, Wang Y, et al. A novel risk score to predict 1-year functional outcome after intracerebral hemorrhage and comparison with existing scores. Crit Care. 2013;17(6):R275-R.
12. Kidwell CS, Rosand J, Norato G, Dixon S, Worrall BB, James ML, et al. Ischemic lesions, blood pressure dysregulation, and poor outcomes in intracerebral hemorrhage. Neurology. 2017;88(8):782-8.
13. Law ZK, Ali A, Krishnan K, Bischoff A, Appleton JP, Scutt P, et al. Noncontrast Computed Tomography Signs as Predictors of Hematoma Expansion, Clinical Outcome, and Response to Tranexamic Acid in Acute Intracerebral Hemorrhage. Stroke. 2020;51(1):121-8.
14. Leasure AC, Sheth KN, Comeau M, Aldridge C, Worrall BB, Vashkevich A, et al. Identification and Validation of Hematoma Volume Cutoffs in Spontaneous, Supratentorial Deep Intracerebral Hemorrhage. Stroke. 2019;50(8):2044-9.

15. Li Q, Liu QJ, Yang WS, Wang XC, Zhao LB, Xiong X, et al. Island Sign: An Imaging Predictor for Early Hematoma Expansion and Poor Outcome in Patients With Intracerebral Hemorrhage. Stroke. 2017;48(11):3019-25.
16. Li Q, Yang WS, Chen SL, Lv FR, Lv FJ, Hu X, et al. Black Hole Sign Predicts Poor Outcome in Patients with Intracerebral Hemorrhage. Cerebrovascular diseases. 2018;45(1-2):48-53.
17. Miyares LC, Falcone GJ, Leasure A, Adeoye O, Shi F-D, Kittner SJ, et al. Race/ethnicity influences outcomes in young adults with supratentorial intracerebral hemorrhage. Neurology. 2020;94(12):e1271-e80.
18. Palm F, Henschke N, Wolf J, Zimmer K, Safer A, Schröder RJ, et al. Intracerebral haemorrhage in a population-based stroke registry (LuSSt): incidence, aetiology, functional outcome and mortality. J Neurol. 2013;260(10):2541-50.
19. Qiu M, Sato S, Zheng D, Wang X, Carcel C, Hirakawa Y, et al. Admission Heart Rate Predicts Poor Outcomes in Acute Intracerebral Hemorrhage: The Intensive Blood Pressure Reduction in Acute Cerebral Hemorrhage Trial Studies. Stroke. 2016;47(6):1479-85.
20. Rådholm K, Arima H, Lindley RI, Wang J, Tzourio C, Robinson T, et al. Older age is a strong predictor for poor outcome in intracerebral haemorrhage: the INTERACT2 study. Age and ageing. 2015;44(3):422-7.
21. Rodriguez-Luna D, Rubiera M, Ribo M, Coscojuela P, Pagola J, Piñeiro S, et al. Serum low-density lipoprotein cholesterol level predicts hematoma growth and clinical outcome after acute intracerebral hemorrhage. Stroke. 2011;42(9):2447-52.
22. Rodriguez-Luna D, Coscojuela P, Rubiera M, Hill MD, Dowlatshahi D, Aviv RI, et al. Ultraearly hematoma growth in active intracerebral hemorrhage. Neurology. 2016;87(4):357-64.
23. Roeder SS, Sprügel MI, Sembill JA, Giede-Jeppe A, Macha K, Madžar D, et al. Influence of the Extent of Intraventricular Hemorrhage on Functional Outcome and Mortality in Intracerebral Hemorrhage. Cerebrovascular diseases. 2019;47(5-6):245-52.
24. Sato S, Koga M, Yamagami H, Okuda S, Okada Y, Kimura K, et al. Conjugate eye deviation in acute intracerebral hemorrhage: stroke acute management with urgent risk-factor assessment and improvement--ICH (SAMURAI-ICH) study. Stroke. 2012;43(11):2898-903.
25. Sato S, Delcourt C, Zhang S, Arima H, Heeley E, Zheng D, et al. Determinants and Prognostic Significance of Hematoma Sedimentation Levels in Acute Intracerebral Hemorrhage. Cerebrovascular diseases. 2016;41(1-2):80-6.
26. Saxena A, Anderson CS, Wang X, Sato S, Arima H, Chan E, et al. Prognostic Significance of Hyperglycemia in Acute Intracerebral Hemorrhage: The INTERACT2 Study. Stroke. 2016;47(3):682-8.
27. Siddiqui FM, Langefeld CD, Moomaw CJ, Comeau ME, Sekar P, Rosand J, et al. Use of Statins and Outcomes in Intracerebral Hemorrhage Patients. Stroke. 2017;48(8):2098-104.
28. Sun S, Pan Y, Zhao X, Liu L, Li H, He Y, et al. Prognostic Value of Admission Blood Glucose in Diabetic and Non-diabetic Patients with Intracerebral Hemorrhage. Scientific reports. 2016;6:32342-.
29. Yu S, Arima H, Heeley E, Delcourt C, Krause M, Peng B, et al. White blood cell count and clinical outcomes after intracerebral hemorrhage: The INTERACT2 trial. J Neurol Sci. 2016;361:112-6.

30. Zheng D, Sato S, Arima H, Heeley E, Delcourt C, Cao Y, et al. Estimated GFR and the Effect of Intensive Blood Pressure Lowering After Acute Intracerebral Hemorrhage. American Journal of Kidney Diseases. 2016;68(1):94-102.
